# Modern Sociology

**Published:** 1902-04-12

**Authors:** 


### Modern Sociology.

### SPECIAL SCHOOLS UNDER THE LONDON SCHOOL BOARD.

### III.—Schools for the Feeble-Minded.

Between the normal and the idiot child there is not only a great gulf fixed, but there are varying degrees of mental capacity, which may be roughly grouped under the heads "feeble-minded" and "imbecile." At present there is no organisation for the imbecile child, for whom the starting of custodial homes is greatly needed; this is evident when one reads of the refusal, between March, 1900, and March, 1901, of 128 children who were too stupid to be admitted to the classes for special instruction of the feeble-minded. Of children thus refused, few, if any, find admission into the Poor-Law Schools; and if their parents take the trouble to inquire they are frequently told that there is no room in the homes or asylums. Even after the admission of doubtful cases it was found necessary, during the period referred to, to dismiss 47 boys and 19 girls, after a fair trial, as "uneducable," so that in the twelve months ending last March 197 children were certified by the medical officer of the Board as unfit. "Surely," adds the report which gives these figures, "it is wiser economy to make provision for their permanent care than that they should have liberty to multiply their number."

Up till 1891 there was no special provision for feeble-minded children under the School Board, and the first attempt to deal with the problem was made in that year, the Royal Commission of 1889 having recommended that the feeble-minded should be separated from the ordinary scholars in public elementary schools, in order that they might receive special instruction. Two special schools were opened in 1892, and Mrs. Burgwin, who had led the agitation against over-pressure, was appointed superintendent. These schools were for "children who by reason of physical or mental deficiency could not be taught in the ordinary standards or by ordinary methods." By 1898 it was found that one per cent. of the children between 7 and 14 needed special instruction, and it is largely owing to Mrs. Burgwin's untiring efforts that these children are now being adequately dealt with.

There are special difficulties attending the work of teaching the feeble-minded child, among which uncertainty of temper (there are times when "nothing," as one teacher expressed it, "goes right for a week") moral depravity, (sometimes developed to an appalling degree), lack of memory and will power, and general apathy, play a large part. Against these the teacher has to contend by all possible means. The work has, however, its hopeful side, and it has been found that the children improve to a remarkable degree under the care of a painstaking teacher. At a school in Dulwich, when the children first came the teacher tried in vain to attract their attention by changing the flowers in the windows every day. For some time absolutely no impression seemed to be made, but now, after 18 months' conscientious work, the pupils take the greatest delight in bringing flowers, leaves, and berries, and in watching the oak-tree develop slowly from the acorn they themselves have picked up during a walk in Brockwell Park. "We begin," says Mrs. Burgwin, "with the rudimentary idea of self-respect; we train the children in habits of cleanliness and politeness, even before we begin to try and develop their powers of observation." During a visit to the school just mentioned a boy was pointed out to us as having come to this special school as a last chance. He had been punished for truancy at the ordinary school over and over again, had been before the magistrate, and had, as the teacher said, "done more wicked things than could be mentioned." He looked heavy and sullen, but he came to school of his own free-will, no special restriction being made except that he was not allowed to mix with the other children in the playground. Yet over this heavy, unattractive, and stupid face there came quite a pleased and surprised expression when a little trouble was taken with him. It was found that he loved drawing, and was quite interested when his mistake of trying to show both full face and profile at the same time was pointed out to him, or the proportionate size of a gigantic fish to the man supposed to be catching him by a slender line from the bank. It was perhaps the first time any interest had been evinced in his queer artistic productions, and it gave him a feeling that there was something he could do. That feeling is what the teachers try to develop, and directly the thing produced can be made of any practical use—a knitted duster, for example—the child begins to gain a self-respect which it has never known before, and upon which the teacher builds. The instruction is, of course, very elementary, and, except for the age of the children, one might imagine oneself in an infant school. But probably few infants would evince the same curiosity about their lessons as occasionally these "dull" children show. Such words, for instance, as *some* and *sum*, which the average child accepts with resignation, are occasionally severely criticised by the feeble-minded, who wants to know "Why the man made it like that?" Sound, however, plays the largest part in reading lessons, and the words are built up, as in the kindergarten system, from simple combinations. One teacher uses differently coloured chalks for this, so that the eye as well as the ear may receive the impression. Singing simple action songs and the performance of simple physical drill play an important part, but it is evident that more might be done in the way of gymnastics if more space were available. Drawing, as in the case of deaf children, seems to come easily to some of the feeble-minded; and one very weak boy, who hung his head and could hardly be induced to look his teacher in the face, was never tired of drawing the "Twopenny Tube" in perspective. The Dulwich children are being taught to draw, from the shoulder, bold outlines of leaves, eggs, and other simple objects; and this obviates the indolent habit, when the object is small enough, of laying it on the slate and drawing round it—a habit dear to ordinary children as well as to the feeble-minded. The strain on the teacher involved by such work is no light one, and it is not surprising if "Another day over!" represents the state of mind in which a woman thus engaged sometimes finds herself by four o'clock in the afternoon. The work has, however, its keen interest; and the remark of one teacher, that some simple clinical instruction would be of great use, shows the serious way in which it is regarded. The remark was *à propos* of a child whom the doctor had pronounced to be cretinoid, and who had remarkably improved. By allowing special visits to London parks or to the Zoological Gardens to count as school-time, the Board gives valuable opportunities for instruction "from the object" which would not otherwise be possible, and it is evident that this elasticity is very greatly appreciated by the teachers.

At Clerkenwell an interesting experiment is now being tried, where some twenty children, boarded out under the Metropolitan Asylums Board, are daily attending the Hugh Myddelton Special School. It is hoped that the effect may be beneficial on both classes of children; the experiment is, however, too new for any expression of opinion as to results.

It is felt that the missing link in work for the feeble-minded should be supplied by custodial homes, to which the children might go between leaving school at sixteen (the Board's limit) and going to work or entering a permanent institution. The danger at present is that they will drift back, for want of proper care and encouragement, into the condition of apathy from which, by great labour and expense, they have been rescued. These homes for "after-care" should be under the control of the L.C.C. cr the Metropolitan Asylums Board, and "after-care committees" are being formed in view of the need. These are voluntary agencies, formed of ladies interested in the employment of the feeble-minded. The results may to some seem disproportionate to the labour and expense; but if by any means the weak-minded or low-grade child can be turned into a self-guiding person, with due regard for the rights of others, an inestimable service will be rendered to the State.

